# Placenta Percreta in the Absence of a Previous Uterine Scar

**DOI:** 10.7759/cureus.94550

**Published:** 2025-10-14

**Authors:** Nikolaos Antonakopoulos, Apostolos Kaponis, Georgios Katsougiannopoulos, Panagiota Tzela, Georgios Adonakis

**Affiliations:** 1 Department of Obstetrics and Gynecology, School of Health Sciences, University of Patras, Patras, GRC; 2 Department of Midwifery, School of Health and Care Sciences, University of West Attica, Athens, GRC

**Keywords:** beta-thalassemia, deciduo-myometrial interface, dehisced uterine scar, inadequate decidualization, maternal morbidity, placenta accreta spectrum, placenta previa, previous cesarean delivery, severe hemorrhage

## Abstract

Placenta accreta spectrum (PAS) encompasses abnormal placental adherence to the myometrium, ranging from accreta to percreta, and is a major cause of life-threatening hemorrhage. While prior cesarean section and placenta previa are the main risk factors, PAS in the absence of uterine scarring is rare. We report two cases of PAS without prior uterine surgery. The first involved a 42-year-old woman with beta-thalassemia major and a history of cesarean section, who conceived via in vitro fertilization (IVF). She presented at 30 weeks with acute abdominal pain, hypovolemic shock, and fetal demise. Emergency laparotomy revealed posterior uterine rupture and placenta percreta. Despite a hysterectomy and repeated interventions, the patient died due to uncontrolled hemorrhage and coagulopathy. The second case involved a multiparous woman with no surgical history, who presented at 32 weeks with severe bleeding and fetal distress. Intraoperatively, an anterior placenta percreta invading through the uterine serosa was found. A hysterectomy was performed with a favorable maternal outcome. These cases highlight PAS occurrence in women without prior uterine scars, suggesting that additional factors such as beta-thalassemia, chronic hypoxia, oxidative stress, and altered angiogenesis may contribute to abnormal placental invasion. The first case strongly suggests a possible association between beta-thalassemia and PAS. Spontaneous uterine rupture due to unrecognized PAS underscores the need for vigilance even in low-risk women. PAS can occur without classic risk factors and may be linked to systemic conditions such as beta-thalassemia. Early recognition, multidisciplinary management, and timely hysterectomy are crucial to improving maternal outcomes. Further studies are warranted to clarify the role of beta-thalassemia and other non-surgical risk factors in PAS pathogenesis.

## Introduction

Placenta accreta spectrum (PAS) is a general and evolving nomenclature used to describe cases where the anchoring placental villi attach pathologically to, penetrate into, or even pass through the myometrium, reaching the uterine serosa or adjacent organs [1-4]. PAS is classified into three grades based on histologic and/or clinical features: placenta accreta, in which the anchoring villi attach partially to the myometrium and not to the decidua only; placenta increta, in which the anchoring villi penetrate deeply into the myometrium; and placenta percreta, in which the anchoring villi penetrate through the myometrium to the uterine serosa or adjacent pelvic tissues and organs [1]. The exact pathogenetic mechanism remains uncertain, and various theories have been proposed.

Literature data increasingly support the idea that inadequate decidualization in preexisting defects of the deciduo-myometrial interface, particularly involving a dehisced uterine scar, is the key factor [5-7]. Several risk factors for these defects have been reported, with previous cesarean delivery being the most common, especially when it coexists with placenta previa [8,9]. The higher the number of cesarean sections, the greater the likelihood of PAS, with women having more than four scars showing a 10 times higher risk than those with only one scar [10]. This explains the marked increase in PAS prevalence worldwide, which can be traced to the rising number of cesarean births in recent decades [11-13]. Cesarean scar pregnancy (CSP) and PAS likely represent a spectrum of the same disease, as they share similar histological features [9]. Other interventions potentially distorting the deciduo-myometrial interface, such as myomectomy, endometrial ablation, or dilation and curettage, have also been recognized as contributing factors [8,9,14,15].

Interestingly, additional risk factors not related to previous uterine scar have been reported in the literature, such as older maternal age, pregnancy after assisted reproduction techniques, smoking, and beta-thalassemia, although the latter is a rare factor [16-21]. Given the variety of risk factors and the increased maternal and neonatal morbidity associated with PAS, primarily due to severe hemorrhage events, accurate antenatal diagnosis is essential and prognosis is determinant [22,23].

## Case presentation

Case 1

The first case was a posterior high placenta percreta in the absence of a previous scar in the posterior uterine wall. The woman, a 42-year-old Caucasian, was known to have beta-thalassemia, and she was totally dependent upon blood transfusions throughout her life. She already had one child (previous cesarean section), but she opted for an IVF pregnancy due to her new husband. The woman was admitted to the emergency department of our hospital with painful uterine bleeding at 30 weeks of gestation and collapsed soon after her admission. The woman was complaining of severe pelvic pain at home for approximately two hours prior to her admission. Resuscitation measures were applied immediately, and after the woman partially responded, a harsh abdominal ultrasound revealed a singleton pregnancy with fetal demise and a massive collection of blood posterior to the placenta without a clear contour of the posterior uterine wall. The laboratory blood results revealed massive hemorrhage, and the woman was in hypovolemic shock. The differential diagnosis included placental abruption or uterine rupture.

**Figure 1:**
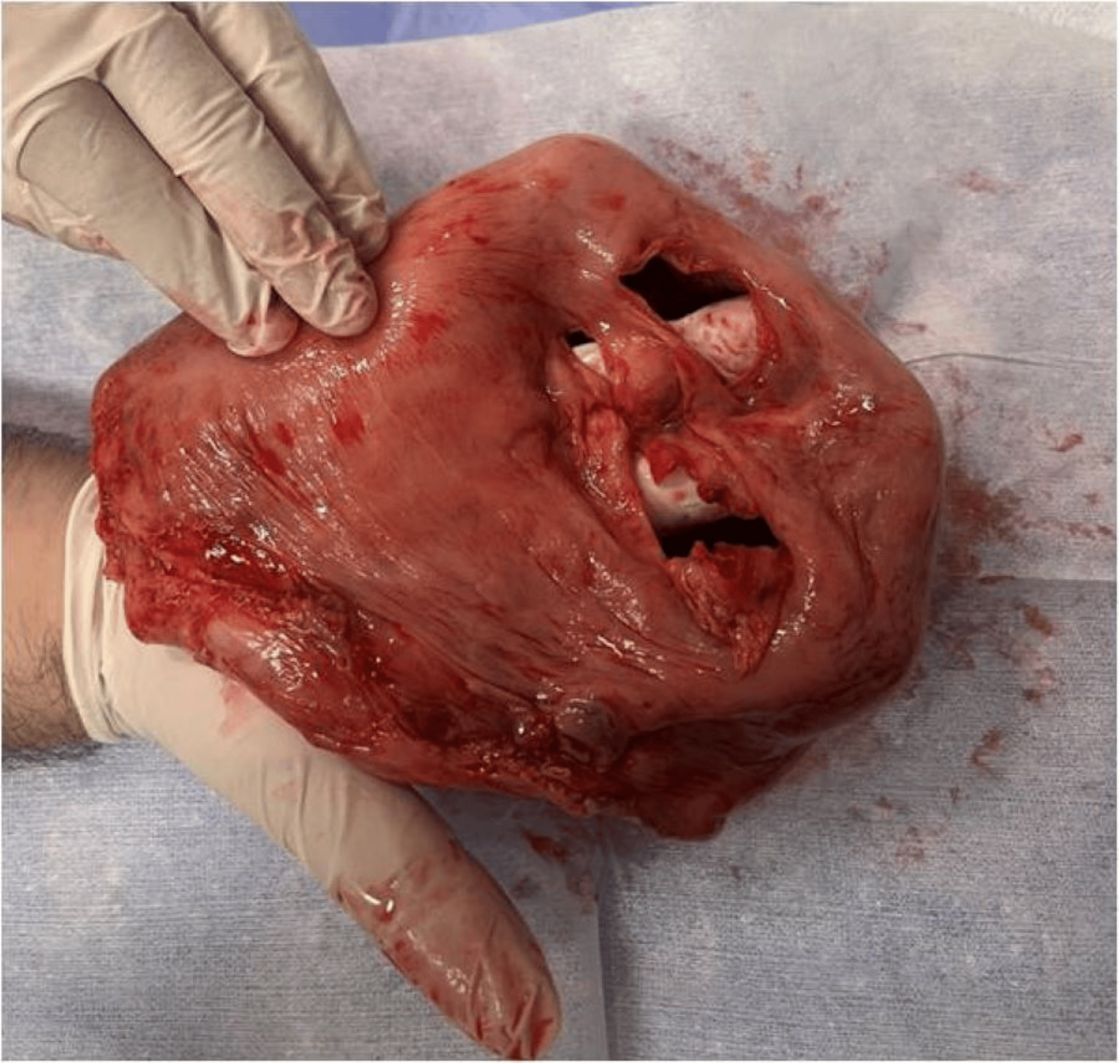
The large posterior uterine wall deficit with extreme thinning of the surrounding uterine wall.

An emergency C-section/laparotomy was performed to control bleeding and increase the woman’s chances of recovering from shock. After cord occlusion, the laborious effort for manual placental separation raised the suspicion of a morbidly adherent placenta. After placental removal, a large uterine wall deficit was found at the posterior uterine wall, with extreme thinning of the surrounding uterine wall (Figure 1). The area of the previous cesarean scar appeared completely normal. There was a minimum hemorrhage during the procedure, due to the woman’s hypovolemia; however, a large amount of blood clots was found and removed from the upper abdominal cavity in the absence of any visceral injury. Given the almost bloodless C-section and the sonographically evident bleeding prior to surgery, the source of these blood clots was attributed to the uterine rupture, which was considered to have occurred prior to the C-section, probably when the woman’s acute symptoms initially manifested. An obstetrical hysterectomy was performed since uterine atony and mild bleeding persisted, in order to prevent further blood loss. After surgery and transfusion of several blood units, the woman was admitted to the ICU for further support.

Due to laboratory and sonographic signs of active intra-abdominal bleeding and clotting disorder, a subsequent laparotomy was performed, 24 hours later, and Logothetopoulos packing was decided as there was generalized uncontrolled bleeding, without a clear source evident, confirming severe secondary coagulopathy. Packing also failed, and pelvic embolization was also offered 24 hours later, as the last resort. Unfortunately, the woman kept deteriorating and died three days after her initial admission.

The macroscopic pathological examination of the uterus confirmed the uterine rupture and extreme thinning of the adjacent uterine wall (thickness less than 1 mm) with placental remnants deeply adherent to the adjacent uterine wall and areas of ischemic necrosis. The microscopic pathological examination revealed necrotic and degenerated placental villi penetrating deeply into the uterine wall, reaching up to 1 mm from the uterine serosa, without intervening decidua. The findings confirmed the diagnosis of morbidly adherent placenta (placenta percreta).

Case 2

**Figure 2:**
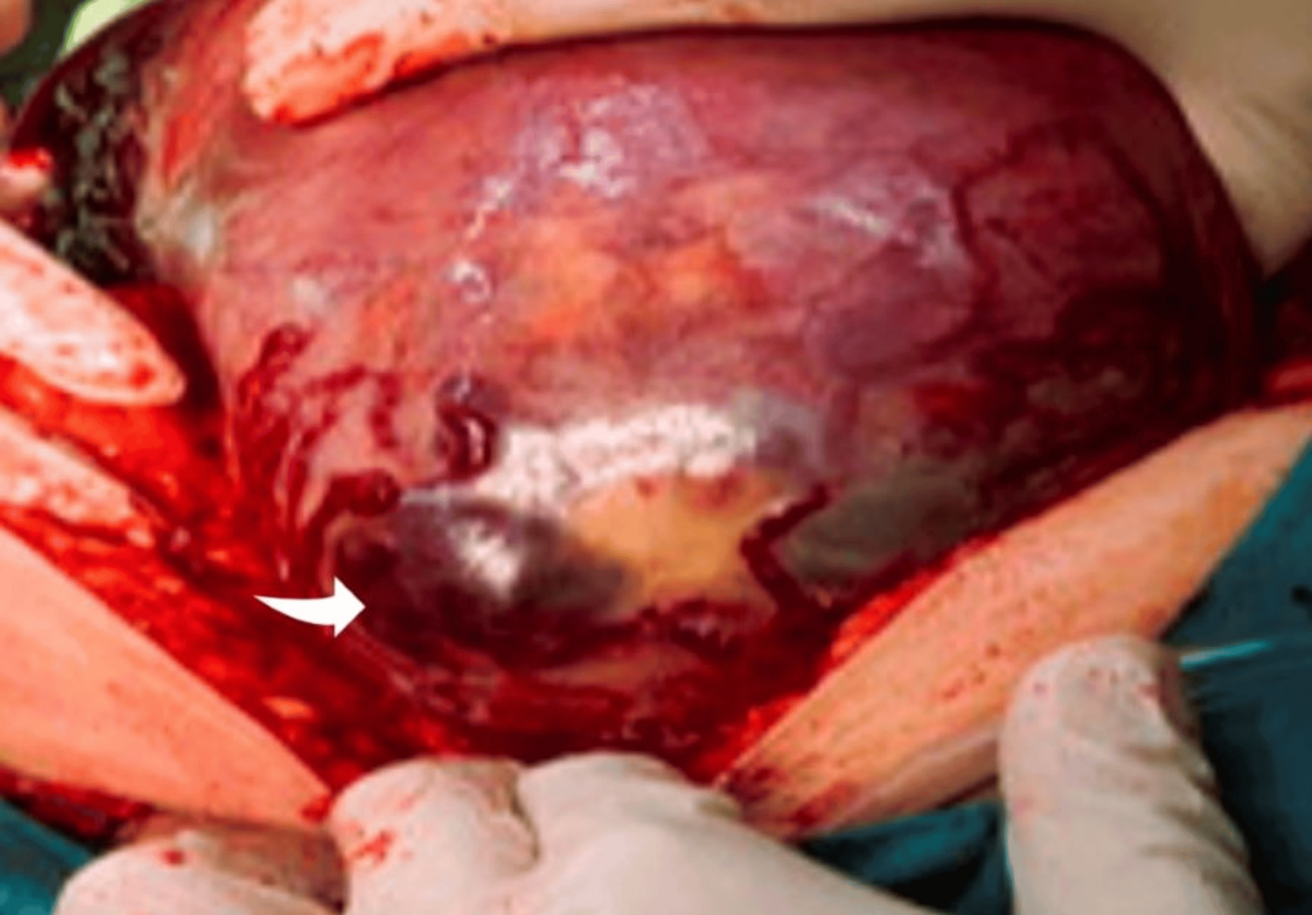
The percreta anterior placenta partially protruding through the uterine serosa (arrow).

The second case (a few months after case 1) was an anterior low placenta percreta without any previous uterine scar, as the woman delivered her first baby vaginally and this was her second pregnancy. This pregnancy, however, was complicated with low-lying anterior placenta, already known since the routine anatomy scan, and cervical insufficiency required cervical cerclage at 16 weeks. The woman was admitted to the emergency department of our hospital due to severe uterine hemorrhage. Since the pregnancy was advanced (32 weeks) and there was evident fetal distress, an emergency cesarean section under general anesthesia was performed. Surprisingly, a massive hemoperitoneum was found, and a percreta anterior placenta partially protruding through the uterine serosa (Figure 2), causing active bleeding in the intraperitoneal cavity. The non-protruding part of the placenta was placenta accreta. The placenta was removed, and the uterine wall deficit was sutured. However, due to persistent bleeding and after failure of other more conservative measures (administration of uterotonics, tranexamic acid, and hemostatic sutures), an obstetrical hysterectomy was performed. The post-operative course of the woman was uneventful.

## Discussion

As already mentioned, the typical scenario of a pregnant woman with PAS is the combination of placenta previa and a history of one or more prior cesarean sections. The frequency of PAS increases with the number of cesarean births from 11% after one cesarean delivery to 67% after four cesarean deliveries. In the absence of placenta previa, the frequency of PAS is lower, estimated to be approximately 0.2% in women with one previous cesarean birth and reaching 4.7% with five or more previous cesarean births. Recent studies support the scenario of the deciduo-myometrium defect in PAS, often associated with the absence of reepithelialization in the scar area. As a result, the myometrium and its vasculature are exposed below the junctional zone to the extravillous migrating trophoblast. Furthermore, excessive vascular remodelling, combined with increased high-velocity blood flow from the abnormal uterine circulation, leads to the formation of placental lacunae and fibrinoid deposition. These changes are correlated with the absence of the Nitabuch membrane and contribute to abnormal placental attachment [24-26].

In our first case, the previous cesarean scar was found to be absolutely normal, without any signs of dehiscence, nor was there any history of surgical procedures that could have contributed to a uterine scar, such as myomectomy or endometrial curettage.

However, even in the absence of a previous cesarean birth, there is a remaining risk of 3% for PAS, and if placenta previa is further excluded, like in our case, the incidence of PAS is extremely low, approximately 0.03%, but not zero [27]. Thus, there must be a mechanism other than uterine scarring potentially leading to PAS, although the root of PAS pathophysiology is considered entirely iatrogenic by the experts in the field [25].

Our first case had no previous intervention that could have caused a uterine scar at the upper posterior uterine wall. The major maternal pathology in our case was thalassemia, so we targeted our literature review to a potential correlation between maternal thalassemia and PAS.

The study of Wu et al. showed significant differences in the incidence of placenta increta, polyhydramnios, and postpartum anemia between the thalassemia group and the non-thalassemia group [20].

A cross-sectional study has found that abnormal angiogenic and growth factor signalling, such as increased VEGF, leads to early pregnancy proliferation of the uterine and pelvic vasculature [28]. On the other hand, there are several reports indicating that patients with beta-thalassemia have elevated growth factors, like VEGF and angiopoietin-1, which are associated with irregular angiogenesis [29,30]. In our case, where there were no prior iatrogenic factors, beta thalassemia and the weekly transfusions are the main risk factors, as they are linked to impaired angiogenesis and potentially to uterine vasculature abnormalities.

Oxidative stress due to iron overload and placental hypoxia caused by maternal anemia seems to provoke the pregnancy complications in cases with thalassemia [31]. The chronic hypoxic status of thalassemia may affect the placental adherence in the decidua and probably induces deeper penetration into the myometrium, while the increased risk for thrombosis due to the chronic hypercoagulable state of thalassemic women could explain the increased risk of placental complications, such as placental abruption [31].

The initial clinical sign of PAS is often a severe, life-threatening hemorrhage that usually occurs during attempted manual placental separation. This is why it is proposed that the morbidly adherent placenta should remain in situ and be removed along with the uterus, if obstetrical hysterectomy is indicated, or the uterine segment that includes the morbidly adherent placental site, if this is clearly defined and the maternal condition allows the preservation of the uterus [32].

Life-threatening hemorrhage can also occur due to uterine rupture, which is most common in the third trimester and is correlated with a thinner uterine lower segment, similar to our case [33]. The woman usually presents severe symptoms, such as acute severe abdominal pain and distension, tachycardia, or hypovolemic shock. Even more, intrauterine fetal demise documented via absent fetal heart activity is inevitable if the rupture takes place in an outpatient setting [33]. Our case presented exactly this way. There was acute, severe abdominal pain at home, and the woman collapsed soon after her admission into the emergency department. Cases of spontaneous uterine rupture due to PAS, in the second trimester, associated with high maternal and fetal mortality and morbidity have also been reported [33-36].

Interestingly, there are studies to suggest that an abnormal placenta is a risk factor for unscarred uterine rupture [37-39]. We strongly believe that in our case, the uterine rupture occurred spontaneously before the C-section and our attempt to separate the placenta. This would explain the acute dull abdominal pain, the hypovolemic shock, the fetal demise, and the large amount of clotted perihepatic blood found in the upper abdominal cavity during the C-section.

While this case suggests a possible link between beta-thalassemia and PAS, it remains an isolated case report, and further reports are needed for confirmation. Beta-thalassemia major, associated with anemia and frequent blood transfusions, should be further investigated as a potential risk factor of PAS. The absence of prior cesarean sections or surgical scars in this patient is unusual for PAS cases, indicating that additional mechanisms, such as chronic hypoxia, oxidative stress, and abnormal angiogenesis related to beta-thalassemia, may be involved. A retrospective study involving a larger cohort of patients with beta-thalassemia is necessary to validate this hypothesis. Future studies should also investigate this association more deeply, utilizing both clinical and histological data to better understand the underlying mechanisms.

## Conclusions

Our cases underscore the importance of heightened clinical vigilance for PAS, even in women without a history of uterine surgery. Particular attention should be given in the presence of a low-lying placenta or coexisting conditions such as beta-thalassemia, which may increase vulnerability to adverse outcomes. The management of PAS, especially when encountered in early pregnancy, remains complex and requires a multidisciplinary approach. Timely recognition and individualized planning are crucial to minimize maternal and fetal morbidity and mortality, particularly in patients with reduced tolerance to blood loss. When conservative measures fail, the decision to proceed with obstetrical hysterectomy should be made promptly, ideally before maternal hemodynamic compromise occurs. Strengthening awareness, early detection strategies, and proactive management protocols will be key to improving outcomes in these challenging cases.
